# Acute Kawasaki disease with emphasis on the echocardiographic profile: A single center experience

**DOI:** 10.21542/gcsp.2017.27

**Published:** 2017-10-31

**Authors:** Hala S. Hamza, Wessam A. Raouf, Asmaa Z. Zaher, Hala M. Agha

**Affiliations:** 1 Department of Pediatrics, Cardiology unit, Cairo University, Cairo, Egypt; 2 Center for Social and Preventive Medicine, Cairo University, Cairo, Egypt

## Abstract

**Background:** Echocardiographic features of acute Kawasaki disease (KD) have not been well characterized in Egyptian children. This study aimed to provide insight into the pattern of cardiac involvement in Egyptian children with Kawasaki disease, focusing on echocardiographic coronary abnormalities and their associated risk predictors.

**Methods and Results:** Medical records of 64 KD patients from 2012 to 2016 were retrospectively analyzed with recalculation of coronary artery z-scores during the first eight weeks after fever onset. All patients received intravenous immunoglobulin (IVIG) and 57.8% were treated within 10 days of illness onset. Coronary abnormalities were found in 53.1% of all patients, and in 43.2% of those who received IVIG within 10 days. Giant aneurysms (z-score>10) comprised 23.5% of all coronary abnormalities. Coronary thrombosis occurred in two patients (5%), both of whom developed myocardial infarction, and one succumbed to heart failure with eventual in-hospital death. Overall, 7% of patients had mitral regurgitation (*n* = 5), 1.5% had aortic regurgitation (*n* = 1), and 7.8% had pericardial effusion (*n* = 5). Among a number of laboratory and clinical predictors, platelet count had the strongest association with coronary abnormalities (Area under Receiver-operating characteristic (ROC) curve: 0.794; 95% confidence interval 0.678–0.910; *P* < 0.001).

**Conclusion:** Coronary abnormalities occur in a substantial percentage of KD in Egypt, with associated evidence of severe inflammation. Further efforts are required to increase awareness of the disease and to emphasize the importance of early IVIG administration. Future studies should also be undertaken to characterize the long term progression profile of the disease as well as the possible genetic background of the disease in Egypt.

## Background

Echocardiography has a key role in the diagnosis, risk stratification and management of patients with Kawasaki disease (KD).^1–3^ Coronary vasculitis, pericarditis, endocarditis and myocarditis can all be encountered upon echocardiographic evaluation in the acute phase of KD. However, coronary involvement appears to have the most detrimental sequelae.^2,4,5^ The American Heart Association (AHA) recommends periodic echocardiographic assessment at the time of diagnosis, at 2 weeks after diagnosis, and at 6–8 weeks, which covers the highest risk period when coronary dilatation, hypercoagulability, and mortality all peak.^1,2^

Coronary abnormalities had been reported in 15% to 25% of children not receiving IVIG, and in 2–6% of those who received IVIG within 10 days of illness.^1–3^

The coronary and cardiac involvement profile of KD in Egyptian children has not been well characterized. This study sought to describe the echocardiographic pattern of acute KD in a large Egyptian pediatric center.

## Patients and methods

### Patient selection

Medical records of patients diagnosed with KD at the Children’s Hospital of Cairo University, between 2012 and 2016 were retrospectively reviewed. Patients meeting the AHA criteria for KD, who had echocardiographic evaluation within the first eight weeks of fever were included.^1^ The study was in line with the institutional ethical policy. Demographic data, clinical features, laboratory results and echocardiographic findings were analyzed. Z-scores for the right coronary artery (RCA), the left anterior descending coronary artery (LAD), and the left main coronary artery (LMCA) were retrospectively calculated using coronary artery internal dimensions and patient’s body surface area measured at the time of the echocardiographic study showing the largest coronary dimensions.^6,7^

Coronary artery dilatation was considered present when any coronary segment exceeded a z-score of 2.5.^1^ Dilatation was classified as small if the z-score was between 2.5 and 5, large between 5 and 10, and giant if more than 10.^8^ Patients were then divided into two groups according to the presence of coronary abnormalities.

### Statistical analysis

Variables were expressed as means with standard deviations (SD), medians with interquartile range (IQR), or counts with percentages, as appropriate. Student t- test or Mann–Whitney U test were used for comparison between groups. Fisher’s exact test and chi-square test were used to compare proportions. Univariable factors that were significantly associated with coronary involvement were included in a multivariate logistic regression analysis to identify variables which were independently associated with coronary involvement. Receiver-operating characteristic (ROC) curve analysis was then employed to determine the area under the curve (AUC) for each significant predictor. Spearman test was used to assess correlations. P values of <0.05 were considered significant. Statistical analysis was performed using the commercially available software IBM SPSS Statistics for Windows (version 19.0. Armonk, NY).

**Figure 1.**
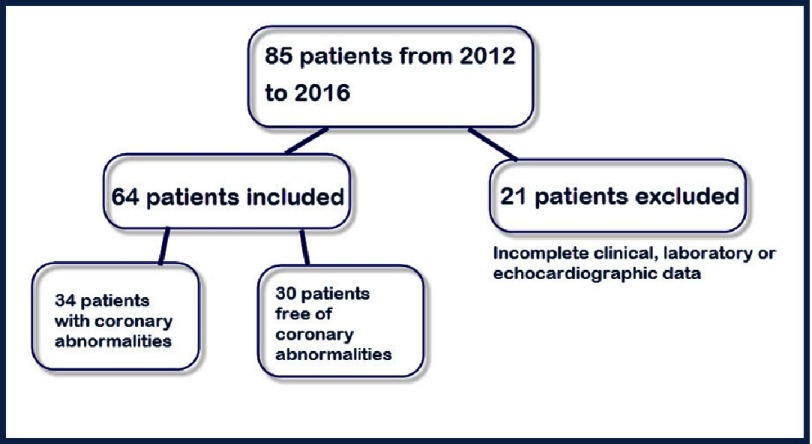
Patient selection.

## Results

Of 85 patients identified by searching the hospital database, 21 were excluded due to incomplete data (Figure 1). The study population comprised of 64 patients, including 23 females (35.9%). All patients had no previous attacks of KD and all received IVIG (2g/kg) with 37 (57.8%) treated within the first 10 days. Five patients received IVIG after the fever had subsided due to either limited access to IVIG where they had been initially diagnosed or delayed search for medical consultation. Coronary arterial lesions were detected in 34/64 (53.1%) of all patients and in 16/37 (43.2%) of those who received IVIG within 10 days.

**Table 1:** The range of coronary abnormalities.

	Right Coronary Artery (*n* = 19)	Left Main Coronary Artery (*n* = 13)	Left Anterior Descending Artery (*n* = 22)
Patients with isolated involvement (n)	8 (42.1%)	3 (23.07%)	9 (40.9%)
Patients with z-score: 2.5-5 (n)	8 (42.1%)	8 (61.5%)	8 (36.36%)
Patients with z-score: 5-10 (n)	6 (31.5%)	4 (30.7%)	8 (36.36%)
Patients with z-score >10 (n)	5 (26.3%)	1 (7.6%)	6 (27.27%)
Thrombus (n)	1	1	2
Diffuse Ectasia (n)	2	1	1

Coronary abnormalities included small coronary aneurysms in 13 (38.2% of all coronary lesions), large coronary aneurysms in 13 (38.2%), giant coronary aneurysms in eight (23.5%). Coronary thrombi were identified in two patients (5% of all coronary abnormalities) with giant aneurysms; both had myocardial infarction, one of them developed depressed myocardial function (shortening fraction: 13.4%) and died in-hospital. None of the patients had coronary stenosis in the acute stage.

The LAD was most commonly affected in 64.7% of patients with coronary involvement, followed by the RCA in 55.8% and the LMCA in 38.2% (Table 1). Measurements of the circumflex artery were not included due to lack of normative values in the z-score calculator used for the current study; however, circumflex dilatation (luminal diameter >3 mm in children less than 5 years old, or >4 mm in children older than 5 years old)^9^ was reported only in two patients who had coexisting LAD and LMCA dilatation. Valve lesions were exclusively found in patients with coronary involvement. Five patients had mitral regurgitation and only one patient had aortic regurgitation. Valvular lesions were mild in degree in all patients except one who had severe mitral regurgitation associated with LMCA and LAD coronary thrombosis and myocardial infarction. Small pericardial effusion was found in five patients, three with coronary abnormalities and two with normal coronaries. Left ventricular indexed dimensions and fractional shortening were not significantly different between patients with or without coronary involvement (Tables 2 and 3). Compared with patients who had no coronary involvement, those with coronary abnormalities had significantly lower hematocrit and hemoglobin levels, higher erythrocyte sedimentation rate (ESR), total leukocyte and platelet counts, longer duration of fever, and more delayed administration of IVIG.

**Table 2:** Clinical criteria of Kawasaki disease patients with and without coronary abnormalities. Data presented as: number (percentage).

	Patients with coronary abnormalities (*n* = 34)	Patients without coronary abnormalities (*n* = 30)
Exanthema (n)	24 (70.5%)	20 (66.6%)
Conjunctival injection (n)	26 (76.4%)	27 (90%)
Changes in Extremities (n)	23 (67.6%)	30 (100%)
Cervical Lymphadenopathy (n)	25 (73.5%)	24 (80%)
Lips & Oral Changes (n)	23 (67.6%)	25 (83.3%)
Refractory KD (n)	4 (11.7%)	3 (10%)
Complete KD (n)	23 (67.6%)	30 (100%)

**Table 3:** Characteristics of patients with and without coronary abnormalities. A *P* value of <0.05 was considered significant.

	Patients with coronary abnormalities (*n* = 34)	Patients without coronary abnormalities (*n* = 30)	*P*-values
Age (years)^a^	3 (1.5-6)	3.25 (2–5)	0.840
Females (n)^b^	10 (29.4%)	13 (43.3%)	0.160
Body surface area (m^2^)^a^	0.61 (0.48–0.74)	0.66 (0.49–0.74)	0.466
Days of fever (n)^a^	14 (9.5–15)	7 (7–12.5)	0.008
Days at IVIG administration (n)^a^	12 (7.75–15.25)	7 (5–12.5)	0.006
Hemoglobin (g/dl)^a^	9.35 (8.6–11)	11 (10–12.1)	0.007
Hematocrit (%)^a^	29.95 (27-31.85)	32 (31.1–32.25)	0.004
PLC (×10^3^/mm^3^)^a^	570.5 (445.5–707.5)	301 (250.7-506)	<0.001
ESR (mm/hour)^a^	104.5 (94.5-120)	60 (53–110)	0.010
TLC (×10^3^/mm^3^)^a^	11.5 (9.6–15)	8.2 (6–12.9)	0.021
Pericardial Effusion^b^	3 (8%)	2 (6%)	
Mitral/Aortic Regurgitation^b^	6 (17%)	0	
LVEDDI (mm/m^2^)^c^	58.2 ± 15.13	52.4 ± 11.3	0.056
LVESDI (mm/m^2^)^c^	35.5 ± 11.47	31.1 ± 7.9	0.071
LVFS%^c^	39.2 ± 0.08	40.7 ± 6 (29–54)	0.580

**Notes.**

a median (interquartile range).

b number (percentage).

c mean ± standard deviations.

ESR erythrocyte sedimentation rate IVIG intravenous immunoglobulin LVEDDI left-ventricular end-diastolic dimension index LVESDI left-ventricular end-systolic dimension index LVFS left-ventricular fractional shortening PLC platelet count TLC total leukocyte count

Among all these variables, platelet count (PLC) performed as the best predictor of coronary involvement (Table 4), (AUC: 0.794; 95% confidence interval 0.678–0.910; *P* < 0.001). Positive correlations were statistically significant between all three individual coronary artery z-scores and day of illness at IVIG administration, ESR, and platelet count. A significant negative correlation was found with hemoglobin level (Table 5).

**Table 4:** Multivariate analysis of factors associated with coronary abnormalities.

	Odds Ratio	95% confidence interval	*P*-value
Hematocrit	0.904	0.611-1.336	0.612
Hemoglobin	1.004	0.437-2.306	0.992
Total leukocytic count	1.068	0.907-1.257	0.429
Platelet count	1.004	1.000-1.008	0.035
Duration of fever	0.995	0.872-1.136	0.944
Intavenous Immunoglobulin administration day	1.016	0.884-1.168	0.821
Erythrocyte sedimentation rate	1.007	0.987-1.028	0.480
Presence of refractory Kawasaki	0.361	0.015-8.823	0.532

**Table 5:** Correlations between different parameters and coronary z-scores. A *P* value of <0.05 was considered significant.

	z-scores for the LAD	z-scores for the LMCA	z-scores for the RCA
Days of fever (n)	r:0.162	r:0.303	r:0.431
	P:0.200	P:0.015	*P* < 0.001
Days at IVIG administration (n)	r:0.327	r:0.351	r:0.356
	P:0.008	P:0.004	P:0.004
Hemoglobin (g/dl)	r:-0.423	r:-0.369	r:-0.262
	P:0.001	P:0.003	P:0.036
Hematocrit (%)	r:-0.459	r:-0.459	r:-0.239
	*P* < 0.001	*P* < 0.001	P:0.057
PLC(×10^3^/mm^3^)	r:0.516	r:0.386	r:0.392
	*P* < 0.001	P:0.002	P:0.001
ESR (mm/hour)	r:0.406	r:0.364	r:0.268
	P:0.001	P:0.003	P:0.033
TLC (×10^3^/mm^3^)	r:0.364	r:0.163	r:0.142
	P:0.003	P:0.199	P:0.262

**Notes.**

ESR erythrocyte sedimentation rate IVIG intravenous immunoglobulins PLC platelets count TLC total leukocytic count

## Discussion

The current study reported more males affected with Kawasaki disease than females, the male:female ratio in the current study was 1.78:1, in keeping with the common male gender predilection found in several studies.^2,10,11^

Echocardiographic abnormalities of the coronary arteries were observed in 53.1% of all patients. This high rate may be only partially accounted for by the relatively delayed administration of IVIG, because it is even higher than that previously reported in patients not receiving IVIG within 10 days in many countries.^10,12–14^ Moreover, even in the subgroup of patients who received IVIG within 10 days after fever onset, 43.2% had z-scores >2.5. This is contrary to the observations of other researchers who reported percentages between 2 and 6% in those receiving full dose IVIG within 10 days.^7,12,15,16^ This raises the possibility of an ethnic or a genetic factor among Egyptian children that may predispose to worse outcomes.

Zhang and colleagues also reported coronary involvement in 63.3% of their cohort of patients.^17^ Similar to other studies, the current study found the LAD to be the most commonly involved artery followed by the RCA then the LMCA.^2,3^ Other researchers found a predilection for the LMCA or equal involvement of the RCA and the LAD.^14,18^

Many studies sought to identify criteria associated with higher risk of coronary abnormalities, and accordingly many laboratory, demographic and clinical parameters have been suggested to predict which KD patients at higher risk for severe vasculitis and coronary artery aneurysms.^1,12,19,20^ This was of more importance before the establishment of IVIG as a standard therapy for all KD patients.^1,20^ However, in regions with limited resources an attempt at risk stratification may be of value, especially to determine the frequency of echocardiographic studies needed, to ensure early IVIG administration, and to consider the use of newer immunomodulatory agents.^21^

In the current study, and in contrast to other studies, age, body surface area, male or female sex, and IVIG refractoriness were not found to be significantly associated with coronary abnormalities.^7,17,22–25^ On the other hand, ESR, platelet count, leukocyte count, hemoglobin, hematocrit, duration of fever, and the day of IVIG administration showed significant association with coronary abnormalities. Similar findings for one or more of these parameters have been reported by other researchers.^20,22,24,26,27^ High platelet count had the strongest association and predictive power for coronary abnormalities. Thrombocytosis showed significant association with coronary abnormalities and indicated more severe vasculitis according to other reports.^20,22,28,29^

## Limitations

The relatively small sample size precludes extrapolation of the findings to the general Egyptian population. Retrospective evaluation of echocardiographic measurements carries a high risk of inconsistent methodology. Many laboratory tests that had been shown to predict coronary abnormalities were not included in the analysis because they were not obtained in many patients (e.g., C-reactive protein, serum albumin, hepatic enzymes).

## Conclusion

This study describes the echocardiographic findings of acute KD in the understudied Egyptian population and highlights the elevated rate of coronary abnormalities. Despite the broad adoption of IVIG treatment in recent years, many patients still experienced delayed administration of IVIG, emphasizing the need for a nation-wide program for early recognition and treatment of KD in Egypt. A genetic variation unique to the Egyptian population should be also sought in the future. In addition, a larger follow-up study to further characterize progression and regression of coronary lesions should be performed.

## Financial Support

This research received no specific grant from any funding agency, commercial or not-for-profit sectors.

## Conflicts of Interest

None.
